# Measurable residual disease after venetoclax treatment for relapsed or refractory chronic lymphocytic leukemia in Japan

**DOI:** 10.1007/s12185-025-04149-z

**Published:** 2025-12-28

**Authors:** Hirotaka Matsui, Jun Takizawa, Kensuke Kojima, Tetsuzo Tauchi, Chiaki Ikeda, Yu Aruga, Hiroki Sakamoto, Risa Takenaka, Jinhaeng Park, Tetsuo Morita, Hiromichi Matsushita, Ritsuro Suzuki

**Affiliations:** 1 Department of Laboratory Medicine, National Cancer Center Hospital, 5-1-1 Tsukiji, Chuo-ku, Tokyo, Japan; 2 Department of Medical Oncology and Translational Research, Graduate School of Medical Sciences, Kumamoto University, Kumamoto, Japan; 3 Faculty of Medicine, Niigata University, Niigata, Japan; 4 Department of Hematology, Kochi Medical School, Kochi University, Kochi, Japan; 5 Shin-Yurigaoka General Hospital, Kanagawa, Japan; 6 Sapporo Clinical Laboratory Inc., Hokkaido, Japan; 7 AbbVie GK, Tokyo, Japan; 8 Department of Laboratory Medicine, Keio University School of Medicine, Tokyo, Japan; 9 Faculty of Medicine, Shimane University, Shimane, Japan

**Keywords:** Chronic lymphocytic leukemia, Measurable residual disease, Real world, Venetoclax

## Abstract

Measurable residual disease (MRD), defined as the persistence of minimal levels of leukemic cells following therapeutic intervention, serves as a pivotal prognostic biomarker in patients with chronic lymphocytic leukemia (CLL). We conducted a multicenter, cross-sectional study to assess MRD in Japanese patients with relapsed or refractory CLL. All patients received venetoclax-based therapy for 24 months. MRD in peripheral blood (PB) and bone marrow was assessed by multicolor flow cytometry using surface markers, including kappa and lambda light chains of surface immunoglobulins. The primary and secondary outcomes were the proportions of patients who achieved undetectable MRD (uMRD, < 10^−4^ CLL cells) and low-MRD (< 10^−2^ and ≥ 10^−4^ CLL cells) after 24 months of venetoclax treatment. From June 2023 to December 2024, 60 patients were enrolled, and 51 were analyzed. The median age was 78 years, and 68.6% were male. Thirty-four of 51 patients (66.7%) achieved uMRD, and 8 (15.7%) achieved low-MRD in PB after at least 24 months of venetoclax treatment. Assessment of MRD in PB by flow cytometry is helpful for evaluating treatment response, informing clinical decision-making, and predicting long-term outcomes in clinical practice. Further analyses are warranted to investigate the use of MRD in treatment decision-making and prognostication.Kindly check the inserted city in affiliations 5 and 9.The city names were corrrected.

**Supplementary Information:**

The online version contains supplementary material available at 10.1007/s12185-025-04149-z.

## Introduction

Measurable residual disease (MRD) is the presence of persistent, low-level leukemic cells after treatment, even in patients with no clinically measurable disease [1–3]. In chronic lymphocytic leukemia (CLL), residual leukemia cells often remain even after the achievement of complete remission (CR) [4]. The low MRD levels are associated with long-term progression-free survival (PFS) and overall survival in patients with CLL [5, 6]. Among patients with similar MRD levels, a better outcome is predicted in patients with CR compared with patients with partial remission (PR) [7]. The International Workshop on Chronic Lymphocytic Leukemia (iwCLL) recommends the assessment of MRD in clinical studies aiming to maximize the remission or response rates [3]. Global guidelines and oversea regulatory agencies recommend the use of undetectable MRD (uMRD) as a predictor of outcome in clinical studies [8–12].

The global phase III MURANO study assessed MRD in peripheral blood (PB) from patients with relapsed or refractory (R/R) CLL administered venetoclax with rituximab, and showed that a high proportion of patients treated with venetoclax achieved uMRD status, which is associated with long-term PFS [4]. The median PFS from the end of treatment for patients with uMRD (< 10^−4^ CLL cells, i.e., fewer than 1 CLL cell per 10,000 leukocytes) was 52.5 months vs 18.0 months in those who were MRD+ at the end of treatment [13]. The Japanese Phase I/II M13-834 study of patients with R/R CLL or small lymphocytic lymphoma administered venetoclax with rituximab also evaluated MRD, but only in six patients [14]. Therefore, the present multicenter, observational, cross-sectional study was conducted to assess MRD in Japanese patients with R/R CLL who received venetoclax, with or without rituximab, for up to 24 months.

## Materials and methods

### Study design and participants

The inclusion criteria of the present study were Japanese patients with R/R CLL, age of 18 years or more, and venetoclax administration with or without rituximab for 24 months or more. The exclusion criteria were treatment with other concomitant CLL medications, participation in other clinical studies, and missing venetoclax administration data. Written informed consent was obtained before enrollment in this study. This study was conducted in accordance with the Declaration of Helsinki and the Japanese Ethical Guidelines for Medical and Biological Research Involving Human Subjects. This study was approved by the National Hospital Organization Nagoya Medical Center’s IRB #2, serving as the central ethics review committee, and registered at the University Hospital Medical Information Network Center (UMIN000050967).

### Data collection

The data from enrolled patients were collected in case report forms using an electronic data capture system. The baseline patient demographic and clinical characteristics included age, sex, Eastern Cooperative Oncology Group performance status (ECOG PS), Rai stage, Binet stage, genetic and chromosomal abnormalities, immunophenotype, and absolute lymphocyte count. CLL treatment data included treatment history and ongoing treatment with venetoclax and rituximab. The clinical response at the time of MRD measurement was evaluated by physicians and classified as CR, CR with incomplete bone marrow (BM) recovery (CRi), PR, nodular PR (nPR), stable disease, or progressive disease in accordance with the response criteria of the 2018 iwCLL guidelines [3].

### MRD measurement

The PB samples were collected within 3 months after each patient received 24 months of venetoclax treatment. BM samples were collected for this study if physicians conducted BM aspiration in routine practice. MRD was measured using multicolor flow cytometry (FCM) at the central facility (Sapporo Clinical Laboratory, Inc., Hokkaido, Japan). For FCM, the following 10 cell surface markers were used: CD5, CD19, CD43, CD79b, CD81, CD23, cell surface immunoglobulin (sIg) kappa, sIg lambda, CD200, and ROR1 [15]. For MRD detection, the expression of sIg kappa/lambda was evaluated as previously described [16], with the fraction with kappa/lambda ≥ 3 or lambda/kappa ≥ 2 as a guide. The average number of analyzed white blood cells was 917,874 (range 287,292–1,258,129). uMRD was defined as the detection of < 10^−4^ CLL cells (< 100 CLL cells in 1,000,000 leukocytes), low-MRD (L-MRD) was defined as < 10^–2^ and ≥ 10^−4^ CLL cells, and high-MRD (H-MRD) was defined as ≥ 10^−2^ CLL cells [4, 17]. The primary and secondary outcomes were uMRD and L-MRD rate after venetoclax treatment for 24 months.

### Statistics

The required sample size was estimated to be 49 patients, based on the frequency of uMRD in the MURANO study using the precision of 13.5% and a 95% confidence interval derived from the Wald test [4].

All data were descriptively summarized, with medians and ranges (minimum, maximum) presented for continuous variables, and numbers and proportions of patients presented for categorical variables. The proportion of patients who achieved uMRD was compared between subgroups categorized by CLL treatment using Fisher’s exact test, and the proportion of patients by venetoclax treatment status after 24 months of treatment was compared between subgroups categorized by patient characteristics using the Mann–Whitney U test, with *p* < 0.05 (two-tailed) considered statistically significant. Statistical analyses were performed using SAS version 9.4 (SAS Institute, Cary, NC, USA).

## Results

### Patient disposition and characteristics

**Fig. 1:**
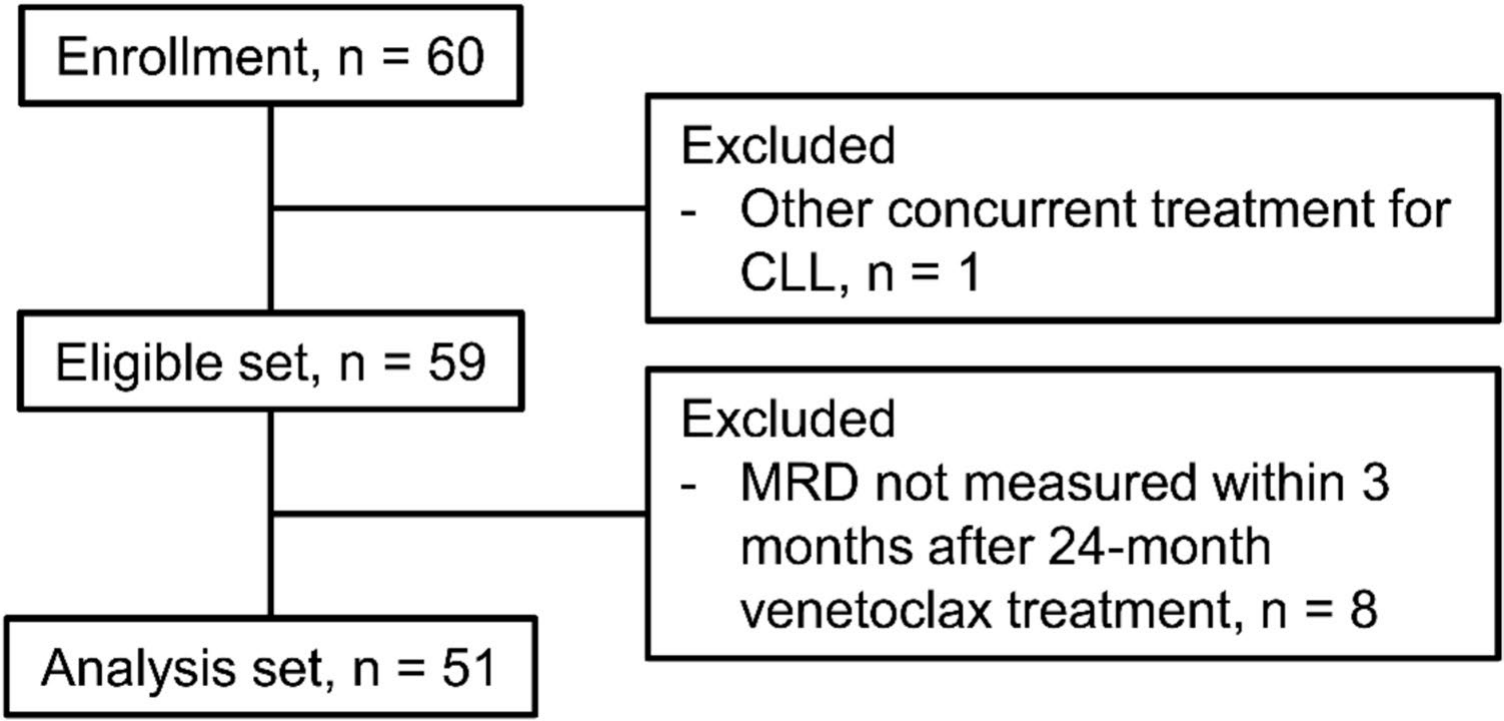
Patients disposition. *CLL* chronic lymphocytic leukemia, *MRD* measurable residual disease

Between June 2023 and December 2024, a total of 60 patients were recruited and included in the intent-to-treat (ITT) population. Of these, 51 patients were considered evaluable. One patient was excluded due to receiving a concomitant CLL treatment, and eight patients were not evaluable because they did not have MRD measured at the required time point (Fig. 1). The median patient age was 78 years, and 35 patients (68.6%) were male (Table 1). More than 80% of patients had at least five markers (CD5, CD19, CD23, sIg kappa, sIg lambda) measurable at baseline in each hospital (Supplementary Table 1).

**Table 1:** Demographic and baseline disease characteristics

Characteristics		n = 51
Sex, n (%)	Male	35 (68.6)
	Female	16 (31.4)
Age, years	Median (range)	78 (49–93)
ECOG performance status, n (%)	0	31 (60.8)
	1	15 (29.4)
	2	4 (7.8)
	3	1 (2.0)
Rai stage, n (%)	0	5 (9.8)
	I	5 (9.8)
	II	7 (13.7)
	III	21 (41.2)
	IV	10 (19.6)
	Unknown	3 (5.9)
Binet stage, n (%)	A	14 (27.5)
	B	11 (21.6)
	C	24 (47.1)
	Unknown	2 (3.9)
17p deletion, n (%)	Present	6 (11.8)
	Absent	29 (56.9)
	Unknown	16 (31.4)
*TP53* mutation, n (%)	Mutated	1 (2.0)
	Unmutated	16 (31.4)
	Unknown	34 (66.7)
*IGHV* mutation, n (%)	Mutated	1 (2.0)
	Unmutated	2 (3.9)
	Unknown	48 (94.1)
Absolute lymphocyte counts per µL	Median (range)	19,855.0 (32.0–206280.0)

*ECOG* European Cooperative Oncology Group, *IGHV* immunoglobulin heavy chain gene

### CLL treatment

The median number of prior CLL treatment lines was two, with patients having received between one and five lines of therapy before enrollment. Twenty-five patients (49.0%) had received one line of treatment (Table 2). The most common recent CLL treatment was the Bruton’s tyrosine kinase (BTK) inhibitor ibrutinib, which was administered to 24 patients.

**Table 2:** CLL treatment status

		n = 51	
Number of prior CLL treatment, n (%)	1	25 (49.0)	
	2	12 (23.5)	
	3	7 (13.7)	
	4	5 (9.8)	
	5	2 (3.9)	
Prior CLL treatment, n (%)		Prior treatment	As the most recent treatment
BTK inhibitors	Acalabrutinib	4 (7.8)	2 (3.9)
	Ibrutinib	38 (74.5)	24 (47.1)
Others	Alemtuzumab	1 (2.0)	1 (2.0)
	Bendamustine hydrochloride	10 (19.6)	3 (5.9)
	Cyclophosphamide monohydrate	18 (35.3)	4 (7.8)
	Fludarabine phosphate	17 (33.3)	2 (3.9)
	Ofatumumab	3 (5.9)	0 (0.0)
	Rituximab	18 (35.3)	5 (9.8)
Daily dose of venetoclax in the maintenance phase, n (%)	400 mg	28 (54.9)	
	≥ 200 and < 400 mg	11 (21.6)	
	< 200 mg	12 (23.5)	
Venetoclax treatment status after 24 months of treatment, n (%)	Completed	27 (52.9)	
	Ongoing	24 (47.1)	
Venetoclax treatment, n (%)	With rituximab	37 (72.5)	
	Without rituximab	14 (28.5)	
Number of rituximab doses	Median (range)	6 (2–21*)	

*BTK* Bruton’s tyrosine kinase, *CLL* chronic lymphocytic leukemia

^*^Only one patient received 21 doses, and the other patients received 6 or fewer doses

Twenty-eight patients (54.9%) received 400 mg of venetoclax daily, and 23 patients (45.1%) received less than 400 mg of venetoclax daily in the maintenance phase. Twenty-seven patients (52.9%) completed 24 months of venetoclax treatment, while 24 patients (47.1%) were ongoing after 24 months of venetoclax treatment. For 37 patients (72.5%) who received venetoclax in combination with rituximab, the median number of rituximab doses was six.

### MRD status

**Fig. 2:**
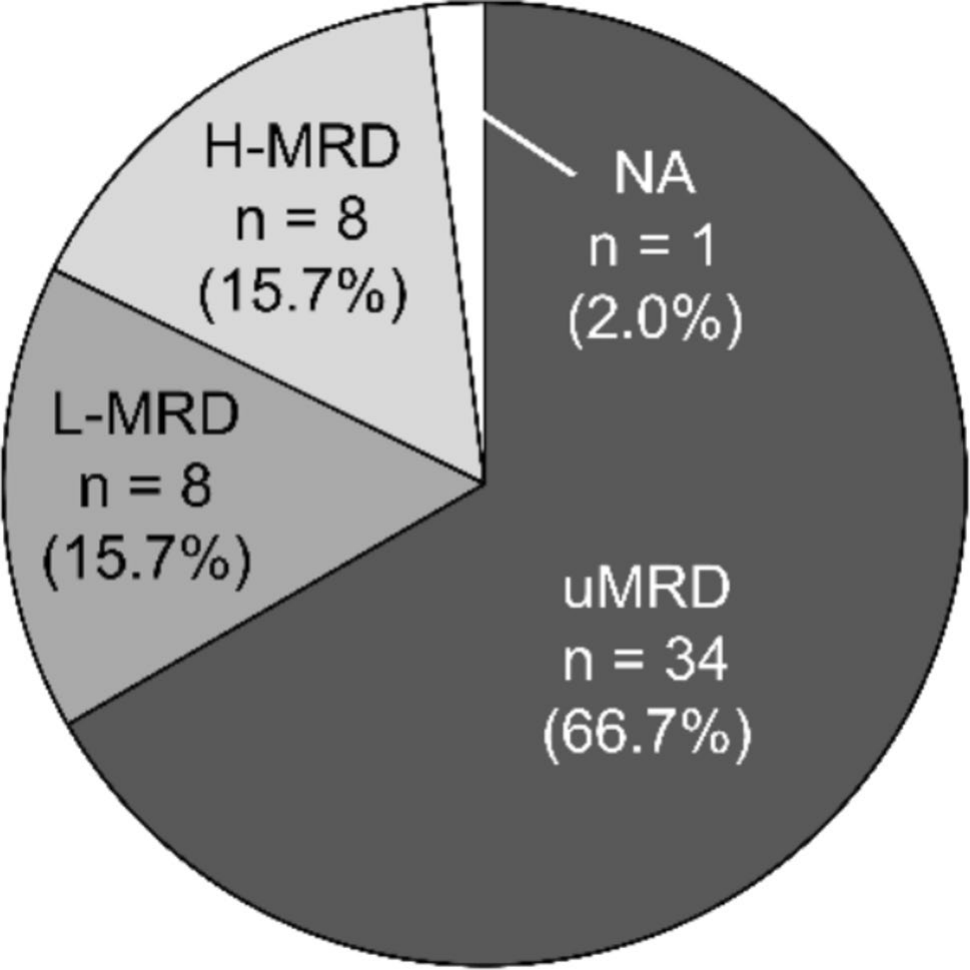
MRD status after 24 months of venetoclax treatment. *H-MRD* high measurable residual disease, *L-MRD* low measurable residual disease, *MRD* measurable residual disease, *NA* not available, *uMRD* undetectable measurable residual disease

The median (range) number of days at which sample collection was performed after the initiation of venetoclax treatment was 775 (687–821) days. The proportions of patients with uMRD, L-MRD, and H-MRD in PB were 66.7% (34 patients), 15.7% (8 patients), and 15.7% (8 patients), respectively (Fig. 2). BM samples were collected from three patients. MRD was detected in only one of those samples. This patient achieved L-MRD in BM and uMRD in PB. MRD status in PB from one patient and in BM from two patients was not determined, because the reverse detection of sIg kappa/lambda ratio showed an inconsistent pattern, occasionally shifting in the opposite direction compared with pretreatment results.

The physician-reported clinical response at the time of MRD measurement was CR/CRi in 86.3% (44 patients; CR 42 patients, CRi 2 patients), PR/nPR in 11.8% (6 patients; PR 5 patients, nPR 1 patient), and stable disease in 2.0% (1 patient) (Table 3). uMRD status was achieved in 30 patients with CR/CRi and 4 patients with PR/nPR.

**Table 3:** Physician-reported clinical response and MRD status at the time of MRD measurement

	n = 51	CR/CRi	PR/nPR	SD
Physician-reported clinical response, n (%)		44 (86.3)	6 (11.8)	1 (2.0)
MRD status, n (%)	uMRD	30 (58.8)	4 (7.8)	0 (0.0)
	L-MRD	8 (15.7)	0 (0.0)	0 (0.0)
	H-MRD	5 (9.8)	2 (3.9)	1 (2.0)
	NA	1 (2.0)	0 (0.0)	0 (0.0)

*CR* complete remission, *CRi* complete remission with incomplete bone marrow recovery, *H-MRD* high measurable residual disease, *L-MRD* low measurable residual disease, *NA* not available, *nPR* nodular partial remission, *PR* partial remission, *SD* stable disease, *uMRD* undetectable measurable residual disease

**Fig. 3:**
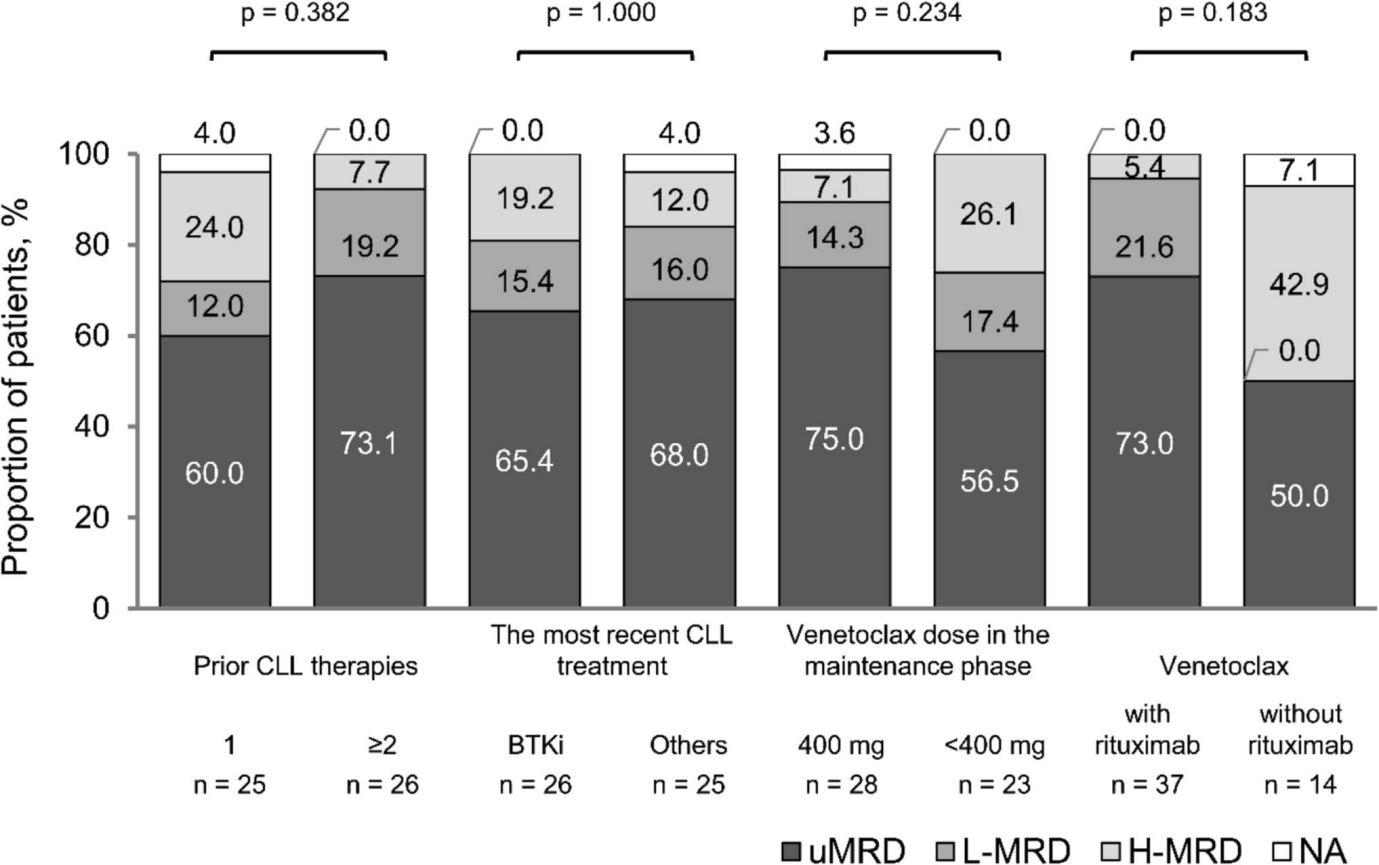
MRD status after 24 months of venetoclax treatment in each subgroup by CLL treatment. *BTKi* Bruton’s tyrosine kinase inhibitor, *CLL* chronic lymphocytic leukemia, *H-MRD* high measurable residual disease, *L-MRD* low measurable residual disease, *NA* not available, *uMRD* undetectable measurable residual disease. P-values were obtained from statistical tests that compared the proportions of patients who achieved uMRD in each subgroup by CLL treatment

MRD rates did not significantly differ between subgroups of patients classified by CLL treatment (Fig. 3). However, the following trends were observed: uMRD rate was higher in patients who received venetoclax in combination with rituximab (73.0%, 27/37 patients) than in patients who received venetoclax monotherapy (50.0%, 7/14 patients) (p = 0.183); and uMRD rate was higher in patients who received 400 mg of venetoclax daily in the maintenance phase (75.0%, 21/28 patients) than in patients who received < 400 mg of venetoclax daily (56.5%, 13/23 patients; ≥ 200 and < 400 mg 54.5%, 6/11 patients, < 200 mg 58.3%, 7/12 patients) (p = 0.234).

The patients who completed venetoclax treatment had lower ECOG PS or received a higher daily dose of venetoclax in the maintenance phase than patients who were receiving ongoing venetoclax after 24 months of treatment (p = 0.025 and 0.078, respectively) (Supplementary Table 2).

## Discussion

We investigated the status of MRD in Japanese patients with R/R CLL after 24 months of venetoclax-based therapy. uMRD rate was 66.7% and L-MRD rate was 15.7% in PB, which were comparable to the results of the MURANO study of patients who received venetoclax in combination with rituximab, in which 83/130 patients (64%) achieved uMRD, and 23/130 patients (18%) achieved L-MRD [4]. The proportion of patients who achieved uMRD was higher among those who received venetoclax in combination with rituximab compared with those who received venetoclax monotherapy (73.0% vs. 50.0%; p = 0.183). The results were consistent with the best MRD responses for venetoclax in combination with rituximab versus venetoclax monotherapy in real-world data from other countries [18, 19]. Patients who achieve uMRD are expected to achieve long-term PFS and overall survival. However, real-world data from other countries have not evaluated differences in MRD status by venetoclax dose. A greater proportion of patients achieved uMRD during the maintenance phase when venetoclax was administered at a daily dose of 400 mg, compared with doses below 400 mg (75.0% vs. 56.5%; p = 0.234), indicating a potential dose–response relationship.

After 24 months of venetoclax treatment, 86.3% (44/51patients) had achieved CR/CRi, and 11.8% (6/51patients) had achieved PR/nPR. The proportion of patients who achieved CR/CRi after venetoclax treatment for 24 months was higher compared with the response rate at 9 months in the MURANO study [20] and at 7.5 months in the M13-834 study [21]. In the MURANO study [22], PFS among venetoclax plus rituximab-treated patients who achieved PR and uMRD at the end of combination therapy was similar to that of patients who achieved CR/CRi. Of the 34 patients with uMRD, 30 were assessed as having a CR/CRi and 4 as having a PR/nPR, based on physician-reported clinical response, and PFS outcomes in both groups may demonstrate comparability.

MRD status in PB was successfully determined in 50 out of 51 patients (98%), using multicolor FCM methods that evaluated cell surface markers, including sIg kappa/lambda expression. BM samples were collected from only three patients because there are few opportunities to collect BM samples in clinical practice. MRD status was not measurable in the BM from two patients from whom BM samples were collected because of the presence of lymphocytes at various maturation stages in the BM. Hematogones, the immature B-cell precursors, are present in the BM and may increase during hematopoietic recovery. At very early developmental stages, they show absent or markedly low expression of surface immunoglobulin light chains and CD20, with these markers increasing as they mature [23]. In addition, hematogones in the BM may occasionally display an apparent light-chain restriction toward either kappa or lambda [23, 24]. Because such findings can make accurate MRD assessment in BM by FCM particularly challenging, MRD results from PB—where feasible—should be interpreted with somewhat greater consideration and compared with the pretreatment tumor immunophenotype whenever possible.

MRD detection can be performed using multicolor FCM, polymerase chain reaction (PCR), or next-generation sequencing [2], and the PCR-based approach has higher sensitivity than the FCM-based approach, but FCM is faster and less laborious [25]. FCM with cell surface markers referenced to the European Research Initiative on CLL (ERIC) [15] which we used, might be the best method to evaluate MRD in PB samples in clinical practice.

The findings of this study indicate that a deep molecular response may be attainable with a 24-month course of venetoclax at a daily maintenance dose of 400 mg in combination with rituximab in patients with relapsed or refractory CLL. The patients who received venetoclax 400 mg as a maintenance dose or in combination with rituximab had higher uMRD rates (75.0% vs. 56.5% and 73.0% vs. 50.0%). However, several limitations must be acknowledged. As described in Supplementary Table 1, cell surface marker information was not available for all patients before or during the treatment; therefore, CLL diagnosis and MRD measurement may be affected. The study cohort was restricted to patients who administered the full 24-month treatment duration, and MRD assessment was conducted only once at the end of treatment, with no interim evaluations. Furthermore, there was no long-term follow-up of clinical outcomes or MRD kinetics, unlike in the MURANO study [4]. Despite these limitations, the application of MRD monitoring in PB by FCM in clinical practice may represent a feasible and valuable strategy for Japanese patients with CLL. Such monitoring could facilitate the assessment of treatment response, support informed decisions regarding treatment duration, and serve as a prognostic tool indicative of long-term disease control.

## Supplementary Information

Below is the link to the electronic supplementary material.

Supplementary file1 (DOCX 45 KB)

## Data Availability

The datasets used and/or analyzed during the current study are available from the corresponding author on reasonable request.
