# Supplementary material for: Measurable residual disease after venetoclax treatment for relapsed or refractory chronic lymphocytic leukemia in Japan
Source: Int J Hematol. 2025 Dec 28;123(5):678–85. doi: 10.1007/s12185-025-04149-z (PMC13171686; doi:10.1007/s12185-025-04149-z)
Supplement: Supplementary file 1 — Supplementary file1 (DOCX 45 KB) [file 12185_2025_4149_MOESM1_ESM.docx]

# Supplementary Tables

## Supplementary Table 1. Physician-reported immunophenotypes of the patients at baseline

| Immunophenotype | | n = 51 |
| --- | --- | --- |
| CD5 | Positive | 44 (86.3) |
|  | Negative | 7 (13.7) |
| CD19 | Positive | 47 (92.2) |
|  | Negative | 2 (3.9) |
| CD43 | Positive | 0 (0.0) |
|  | Negative | 0 (0.0) |
|  | Unknown | 51 (100.0) |
| CD79b | Positive | 4 (7.8) |
|  | Negative | 0 (0.0) |
|  | Unknown | 47 (92.2) |
| CD81 | Positive | 1 (2.0) |
|  | Negative | 0 (0.0) |
|  | Unknown | 50 (98.0) |
| CD23 | Positive | 40 (78.4) |
|  | Negative | 8 (15.7) |
|  | Unknown | 3 (5.9) |
| sIg kappa | Positive | 28 (54.9) |
|  | Negative | 14 (27.5) |
|  | Unknown | 9 (17.6) |
| sIg lambda | Positive | 13 (25.5) |
|  | Negative | 30 (58.8) |
|  | Unknown | 8 (15.7) |
| CD200 | Positive | 6 (11.8) |
|  | Negative | 2 (3.9) |
|  | Unknown | 43 (84.3) |
| ROR1 | Positive | 1 (2.0) |
|  | Negative | 0 (0.0) |
|  | Unknown | 50 (98.0) |

Data are presented as n (%). CD, cluster of differentiation; sIg, surface immunoglobulin; ROR, receptor tyrosine kinase-like orphan receptor.

## Supplementary Table 2. Patient characteristics by venetoclax treatment status after 24 months of treatment

|  |  | Completed,  n = 27 | Ongoing,  n = 24 | P-value  (U test) |
| --- | --- | --- | --- | --- |
| MRD status, n (%) | uMRD | 19 (70.4) | 15 (62.5) | 0.389 |
|  | L-MRD | 4 (14.8) | 4 (16.7) |  |
|  | H-MRD | 3 (11.1) | 5 (20.8) |  |
|  | NA | 1 (3.7) | 0 (0.0) | -* |
| Sex, n (%) | Male | 18 (66.7) | 17 (70.8) | 0.760 |
|  | Female | 9 (33.3) | 7 (29.2) |  |
| Age, years | Median (range) | 79 (49–93) | 77 (60–92) | 0.496 |
| ECOG performance status, n (%) | 0 | 21 (77.8) | 10 (41.7) | 0.025 |
|  | 1 | 3 (11.1) | 12 (50.0) |  |
|  | 2 | 3 (11.1) | 1 (4.2) |  |
|  | 3 | 0 (0.0) | 1 (4.2) |  |
| Rai stage, n (%) | 0 | 4 (14.8) | 1 (4.2) | 0.341 |
|  | I | 3 (11.1) | 2 (8.3) |  |
|  | II | 4 (14.8) | 3 (12.5) |  |
|  | III | 11 (40.7) | 10 (41.7) |  |
|  | IV | 5 (18.5) | 5 (20.8) |  |
|  | Unknown | 0 (0.0) | 3 (12.5) | -* |
| Binet stage, n (%) | A | 8 (29.6) | 6 (25.0) | 0.879 |
|  | B | 6 (22.2) | 5 (20.8) |  |
|  | C | 13 (48.1) | 11 (45.8) |  |
|  | Unknown | 0 (0.0) | 2 (8.3) | -* |
| Number of prior CLL therapies, n (%) | 1 | 12 (44.4) | 13 (54.2) | 0.499 |
|  | ≥2 | 15 (55.6) | 11 (45.8) |  |
| The most recent CLL treatment, n (%) | BTK inhibitor | 16 (59.3) | 10 (41.7) | 0.218 |
|  | Other | 11 (40.7) | 14 (58.3) |  |
| Daily dose of venetoclax in the maintenance phase, n (%) | 400 mg | 18 (66.7) | 10 (41.7) | 0.078 |
|  | <400 mg | 9 (33.3) | 14 (58.3) |  |
| Venetoclax treatment, n (%) | With rituximab | 22 (81.5) | 15 (62.5) | 0.136 |
|  | Without rituximab | 5 (18.5) | 9 (37.5) |  |

BTK, Bruton’s tyrosine kinase; CLL, chronic lymphocytic leukemia; ECOG, Eastern Cooperative Oncology Group; H-MRD, high measurable residual disease; L-MRD, low measurable residual disease; MRD, measurable residual disease; NA, not available; uMRD, undetectable measurable residual disease.

*: Not included in U test
